# Probable levetiracetam-related serum alkaline phosphatase elevation

**DOI:** 10.1186/1471-2377-12-97

**Published:** 2012-09-20

**Authors:** Nian Xiong, Lingling Hou, Na Lu, Asrah A Mohamed, Tao Wang, Yaling Huang

**Affiliations:** 1 Department of Neurology, Union Hospital, Tongji Medical College, Huazhong University of Science and Technology, Hubei 430022, China; 2 Department of Pediatrics, Union Hospital, Tongji Medical College, Huazhong University of Science and Technology, 1277 Jiefang Road, Wuhan, 430022 , Hubei, China; 3 Department of Neurology, General Hospital of Wuhan Iron and Steel (Group) Corporation, 29 Yejin Avenue, Qingshan, Wuhan, Hubei 430080, China

**Keywords:** Levetiracetam, Serum alkaline phosphatase, Hepatotoxicity, Antiepileptic drugs, Epilepsy

## Abstract

**Background:**

Levetiracetam (LEV) is an antiepileptic drug with a favorable tolerability and safety profile with little or no effect on liver function.

**Case presentation:**

Here, we reported an epileptic pediatric patient who developed a significant elevation in serum alkaline phosphatase level (ALP) during LEV monotherapy. Moreover, the serum ALP level was surprisingly decreased to normal after LEV discontinuation. The Naranjo Adverse Drug Reaction Probability Scale score was 6, indicating firstly LEV was a probable cause for the increased serum ALP.

**Conclusions:**

Cautious usage and concerns of the LEV-associated potential ALP elevation should be considered when levetiracetam is prescribed to epilepsy patients, especially pediatric patients.

## Background

Levetiracetam (LEV), structurally similar to the nootropic drug piracetam, is an anticonvulsant medication used for epilepsy treatment. It has been approved by the US Food and Drug Administration as adjunctive therapy for partial seizures in adult and children over 4 years of age with favorable tolerability and safety [1,2]. Moreover, previous studies have been indicated that its efficacy and safety are available in infant and pediatric patients younger than 4 years of age as well [3,4]. It has recently been approved in the European Union as a monotherapy treatment for partial seizures, or as an adjunctive therapy for partial, myoclonic and tonic-clonic seizures [5].

The exactly underlying mechanisms by which LEV exerts its antiepileptic effect have not yet completely understood. Nevertheless, the drug binding to SV2A, a synaptic vesicle protein [6], inhibits nerve conduction across synapses [7]. The common untoward reactions of LEV include asthenia, convulsion, dizziness and somnolence [8]. However, the liver enzyme induction effect of LEV has never been literally reported before. Here, we presented the first case in which elevated serum ALP was highly associated with LEV administration.

## Case presentation

A 10-month-old Han Chinese girl (weight 9 kg) with a history of head trauma was treated conservatively after being diagnosed as intracranial hemorrhage (brain computed tomography scan, brain CT). The patient had been suffered from convulsive attacks (more than 10 episodes/day in the recent 7 days) for 1 week when she was admitted to the Department of Pediatrics, Union Hospital, Tongji Medical College, Huazhong University of Science and Technology. The seizure was characterized as paroxysmal attacks of perioral cyanosis and limbs convulsion, lasting for 2 minutes with spontaneous remission. Her vital signs was stable and within normal limits, and phy-sical and neurological examination revealed no remarkable abnormalities.

**Figure 1:**
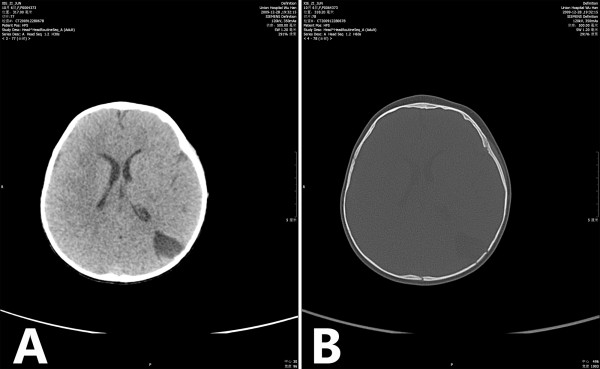
Brain CT. (A) 2.5 × 2.4 cm low density area in left parietal lobe; (B) the interrupted skull continuity in left parietal bone and left occipital.

**Figure 2:**
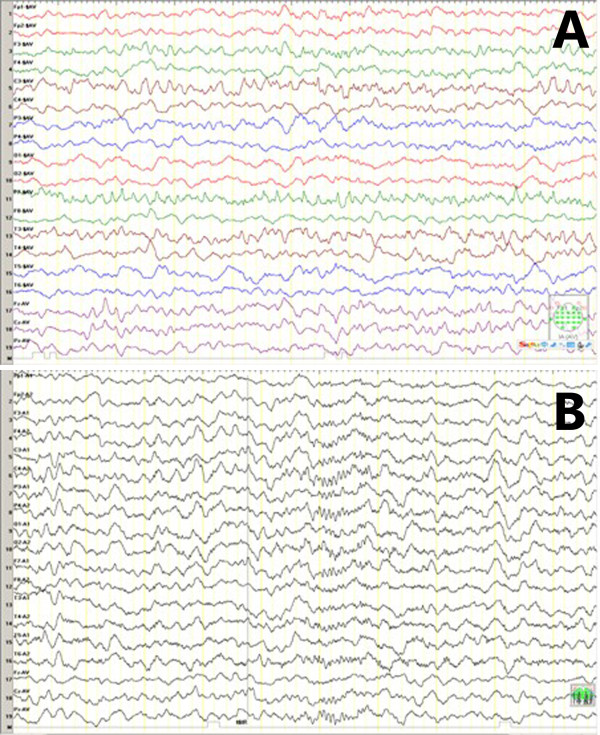
**Routine EEG before and after antiepileptic therapy.** (**A**) Rhythmic spikes in the left central, parietal and temporal areas. (**B**) Normal rhythm of brain waves after antiepileptic treatment.

**Figure 3:**
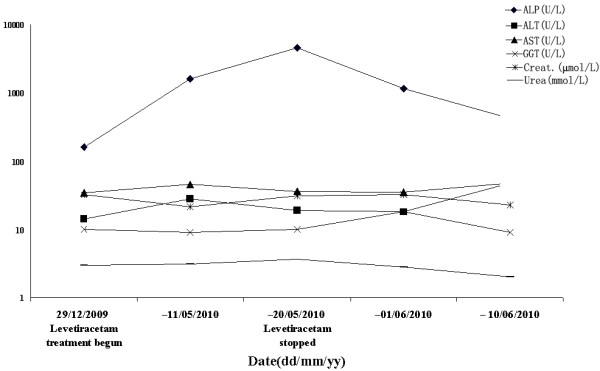
Main indicators of liver function and kidney function before, during and after LEV administration

Brain CT showed left parietal lobe malacia (2.5 × 2.4 cm) and left parietal bone fracture (Figure 1). Meanwhile, the interictal electroencephalogram (EEG) indicated rhythmic spikes in the left central, parietal and temporal areas (Figure 2A). Routine full blood count and blood biochemistry were normal (serum ALP 162 U/L; <500 U/L reference range, Figure 3 and Additional file 1). Based on her disease courses, clinical presentations and auxiliary examinations (CT and EEG), posttraumatic epilepsy was diagnosed. After obtaining informed consent from the patient’s parents, LEV was administrated at an initial dosage of 9.25 mg/kg/d, then 13.8 mg/kg/d, 18.5 mg/kg/d for 1 week, respectively. A final dosage of 27.8 mg/kg/d was prescribed for maintenance therapy. The dose was increased gradually while the patient was seizure-free by the end of the second week after LEV administration.

Five months after LEV treatment, an elevation of serum ALP level (1613 U/L) was accidentally found from a periodical blood biochemistry test without any other abnormalities. One week later, the repeated laboratory test showed that serum ALP level was significantly increased to 4557U/L, accompanied by slightly elevated levels of creatine kinase (81 U/L, 26-140 U/L), creatine kinase-MB activity (50 U/L, 0-24 U/L), lactate dehydrogenase (320 U/L, 109-245 U/L) and bone ALP (>300 U/L, 0-200 U/L). However, no discomfort or seizure activity was observed in this patient. Other related auxiliary examinations included parathyrin level (20.9 pg, 15-68.3 pg), abdominal ultrasound and CT (liver, hepatobiliary system, gallbladder, spleen and kidney), lower extremity long bones X-ray and EEG (Figure 2B) were all normal. Also, no concomitant symptoms and other changes could explain the elevated serum ALP. Therefore, considering the possibility that LEV might be associated with the elevated serum ALP, LEV was discontinued. Surprisingly, the serum ALP level was decreased to 1156 U/L 10 days later. Twenty days after the discontinuation, the patient’s serum ALP level returned to normal (423 U/L). Six months follow-up indicated that the patient was fully recovered, manifesting no recurrent seizure attack or liver function abnormality. She was well developed and nourished during that period, observing from her height, weight, and body mass index.

Naranjo Adverse Drug Reaction Probability Scale [9] was employed to assess the probability correlation of LEV administration and elevated serum ALP. A score of 6 was obtained (Additional file 2), demonstrating LEV was a “probable” cause for the serum ALP elevation, and LEV even possibly led to liver function abnormality although LEV rechallenge was not executed in this patient.

## Discussion and conclusions

Here, we reported a pediatric epilepsy patient who developed elevated serum ALP after LEV treatment. To our knowledge, this is the first report about probable LEV-associated ALP elevation. Before LEV was administrated, the patient’s serum ALP level was normal (162 U/L). The patient was asymptomatic during the intervening period, thus, no other medication was added. As LEV was the only medication for the patient, it was suspected to be the offending agent responsible for the increased ALP level. Moreover, the serum ALP level decreased gradually and returned to normal after the discontinuation of LEV therapy.

The serum ALP activity is commonly measured in clinical chemistry laboratories. Increased values are often associated with disorders of the skeletal or hepatobiliary system, or other possibilities such as leukemia and hyperthyroidism [10]. In our case, all of these potentialities were ruled out by the clinical presentations and auxiliary examination results for parathyrin level, liver, hepatobiliary system, gallbladder, spleen, kidney and lower extremity long bones of this patient. Additionally, history of toxic substances exposure and other drug abuse was absent. Besides, the patient’s increased serum ALP lever was rapidly returned to normal range after discontinuation of LEV, indicating the association between elevated serum ALP and LEV treatment should not be coincidental. Furthermore, the Naranjo Adverse Drug Reaction Probability Scale assessment further confirmed that LEV was a “probable” cause for the serum ALP elevation.

Serious liver function impairment could be rarely observed in patients taking AEDs. Among the traditional AEDs, valproate, which requires routine blood monitoring, can cause hepatotoxicity. Felbamate is the only second-generation AEDs associated with hepatotoxicity that may be fatal. However, liver function impairment has never been reported with LEV. LEV shows rapid absorption, does not bind serum proteins and has linear pharmacokinetics. More than 60% of absorbed LEV can be excreted by the kidneys, approximately 25% is metabolized by metabolized through esterase’s in the blood, less than 3% is plasma protein-bound [11]. Previous study even reported that LEV might affect bone metabolism of both neurologically impaired children and healthy children [12]. Moreover, increased clearance of LEV is reportedly associated with younger age [11]. Although LEV rarely distributes into liver and skeletal sites, nor has displayed any enzyme induction effects in human liver cell tissue [11], it is necessary to present this case and raise the concern of potential hepatotoxicity of LEV.

In summary, we introduced the first case which showed elevated serum ALP level was highly associated with LEV administration. However, it is difficult to confirm the relevance between LEV and serum ALP elevation. Further study is needed extremely to assess the potential hepatotoxicity of LEV in pediatric patients.

## Consent

Written informed consent was obtained from the patient and her parents for publication of this Case report and any accompanying images. A copy of the written consent is available for review by the Series Editor of this journal.

## Abbreviations

LEV: Levetiracetam; AEDs: Antiepileptic drugs; ALP: Alkaline phosphatase; EEG: Electroencephalogram; CT: Computed tomography scan.

## Competing interests

We confirm that we have read the Journal’s position on issues involved in ethical publication and affirm that this report is consistent with those guidelines. None of the authors have any conflict of interest to disclose.

## Authors’ contributions

NX, LH, NL, AM, TW, YH contributed to the conception and design. NX, LH, NL took care of the clinical data collection. NX, LH, AM, TW, YH coordinated all the experiments and helped to draft the manuscript. All authors read, revised and approved the final manuscript.

## Pre-publication history

The pre-publication history for this paper can be accessed here:

http://www.biomedcentral.com/1471-2377/12/97/prepub

## Supplementary Material

Additional file 1 **Biochemistry findings before, during and after LEV treatment.** WBC: white blood cells; RBC: red blood cells; PLT: platelets; ALT: Alanine aminotransferase; AST: aspartate aminotransferase; γGT: gamma glutamyltransferase; Creat: creatinine; CK: creatine kinase; LDH: lactate dehydrogenase. Click here for file

Additional file 2 Adverse drug reactions (ADRs) probability scale. Click here for file
