# Supplementary figures and images for: Probable levetiracetam-related serum alkaline phosphatase elevation
Source: BMC Neurol. 2012 Sep 20;12:97. doi: 10.1186/1471-2377-12-97 (PMC3517503; doi:10.1186/1471-2377-12-97)

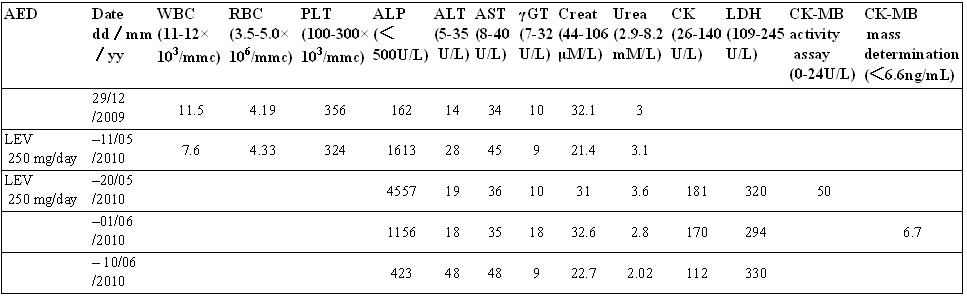

Supplement: Additional file 1 — Biochemistry findings before, during and after LEV treatment. WBC: white blood cells; RBC: red blood cells; PLT: platelets; ALT: Alanine aminotransferase; AST: aspartate aminotransferase; γGT: gamma glutamyltransferase; Creat: creatinine; CK: creatine kinase; LDH: lactate dehydrogenase. [file 1471-2377-12-97-S1.tiff]

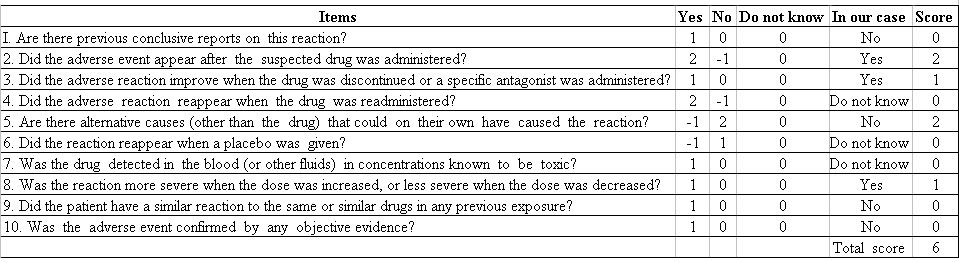

Supplement: Additional file 2 — Adverse drug reactions (ADRs) probability scale. [file 1471-2377-12-97-S2.tiff]
